# Reproducibility of the lung anatomy under active breathing coordinator control: Dosimetric consequences for scanned proton treatments

**DOI:** 10.1002/mp.13195

**Published:** 2018-10-19

**Authors:** Lydia A. den Otter, Evangelia Kaza, Roel G.J. Kierkels, Arturs Meijers, Fred J.F. Ubbels, Martin O. Leach, David J. Collins, Johannes A. Langendijk, Antje‐Christin Knopf

**Affiliations:** ^1^ Department of Radiation Oncology University Medical Center Groningen University of Groningen Groningen 9713 GZ The Netherlands; ^2^ CR‐UK Cancer Imaging Centre The Institute of Cancer Research and The Royal Marsden Hospital London SW7 3RP UK

**Keywords:** active breathing coordinator control, interfraction reproducibility, intrafraction reproducibility, nonsmall‐cell lung cancer, pencil beam scanning

## Abstract

**Purpose:**

The treatment of moving targets with scanned proton beams is challenging. For motion mitigation, an Active Breathing Coordinator (ABC) can be used to assist breath‐holding. The delivery of pencil beam scanning fields often exceeds feasible breath‐hold durations, requiring high breath‐hold reproducibility. We evaluated the robustness of scanned proton therapy against anatomical uncertainties when treating nonsmall‐cell lung cancer (NSCLC) patients during ABC controlled breath‐hold.

**Methods:**

Four subsequent MRIs of five healthy volunteers (3 male, 2 female, age: 25–58, BMI: 19–29) were acquired under ABC controlled breath‐hold during two simulated treatment fractions, providing both intrafractional and interfractional information about breath‐hold reproducibility. Deformation vector fields between these MRIs were used to deform CTs of five NSCLC patients. Per patient, four or five cases with different tumor locations were modeled, simulating a total of 23 NSCLC patients. Robustly optimized (3 and 5 mm setup uncertainty respectively and 3% density perturbation) intensity‐modulated proton plans (IMPT) were created and split into subplans of 20 s duration (assumed breath‐hold duration). A fully fractionated treatment was recalculated on the deformed CTs. For each treatment fraction the deformed CTs representing multiple breath‐hold geometries were alternated to simulate repeated ABC breath‐holding during irradiation. Also a worst‐case scenario was simulated by recalculating the complete treatment plan on the deformed CT scan showing the largest deviation with the first deformed CT scan, introducing a systematic error. Both the fractionated breath‐hold scenario and worst‐case scenario were dosimetrically evaluated.

**Results:**

Looking at the deformation vector fields between the MRIs of the volunteers, up to 8 mm median intra‐ and interfraction displacements (without outliers) were found for all lung segments. The dosimetric evaluation showed a median difference in D_98%_ between the planned and breath‐hold scenarios of −0.1 Gy (range: −4.1 Gy to 2.0 Gy). D_98%_ target coverage was more than 57.0 Gy for 22/23 cases. The D_1 cc_ of the CTV increased for 21/23 simulations, with a median difference of 0.9 Gy (range: −0.3 to 4.6 Gy). For 14/23 simulations the increment was beyond the allowed maximum dose of 63.0 Gy, though remained under 66.0 Gy (110% of the prescribed dose of 60.0 Gy). Organs at risk doses differed little compared to the planned doses (difference in mean doses <0.9 Gy for the heart and lungs, <1.4% difference in V_35_ [%] and V_20_ [%] to the esophagus and lung).

**Conclusions:**

When treating under ABC controlled breath‐hold, robustly optimized IMPT plans show limited dosimetric consequences due to anatomical variations between repeated ABC breath‐holds for most cases. Thus, the combination of robustly optimized IMPT plans and the delivery under ABC controlled breath‐hold presents a safe approach for PBS lung treatments.

## 1 Introduction

Intensity modulated proton therapy (IMPT) using pencil beam scanning (PBS) is a highly conformal radiotherapy technique. Little dose is deposited after the Bragg peak, leading to improved organ at risk (OAR) sparing compared to conventional photon therapy.^1^, ^2^ Furthermore, the intensity modulating properties of IMPT/PBS are superior to passive scattering proton therapy for modulating the dose in and around the target.^1^ However, a major challenge for IMPT/PBS is the treatment of moving targets, such as in nonsmall‐cell lung cancer (NSCLC) patients.^3^ Breathing motion causes “interplay” between the timeline of the proton beam delivery and the timeline of the target motion, leading to misplacement of spots and subsequent unintended dose heterogeneities within the target volume.^4^, ^5^

There are several techniques to mitigate breathing motion and minimize the dose degradation due to the interplay effect. Examples are rescanning,^5^, ^6^, ^7^ tracking,^5^, ^8^, ^9^ gating,^5^, ^10^ and abdominal compression.^11^ The breath‐hold technique is a technique investigated initially for breast cancer patients,^12^ and later also for NSCLC patients.^13^ Here, the radiotherapy treatment is delivered while the patient is holding his/her breath for about 20–25 s, creating a quasi‐static situation. To assist breath‐holding, an Active Breathing Coordinator (ABC) (Elekta Oncology systems Ltd, Crawley, West Sussex, UK) can be used. The ABC device controls the inspiration volume at a given threshold value and aims to produce a reproducible treatment situation. Recently, Kaza et al. investigated the reproducibility of lung volumes with ABC and found lung volume differences by 2% within and by 7% between simulated fractions.^14^ Brock et al. investigated the variability in tumor position when using ABC control for photon therapy in NSCLC patients and found clinically significant tumor movements.^15^ Sarrut et al. investigated the interfraction reproducibility of the lung anatomy using deformable image registration (DIR) on three repeated CT scans during ABC control.^16^ They found ABC control to be effective and reproducible in 6 of 11 patients. Both Brock et al. and Sarrut et al. inspected and evaluated the local displacements, but the dosimetric consequences for IMPT/PBS therapy were not addressed. Dueck et al. investigated the robustness of single‐field uniform dose plans to interfraction uncertainties between repeated voluntary breath‐holds for peripheral located tumors.^13^ Their conclusion was that the smaller tumors and tumors with large baseline shifts were more prone to target coverage loss. In this study, we investigated the anatomical reproducibility of ABC controlled breath‐holding in addition to the work performed by Kaza et al. for both intra‐ and interfraction uncertainties. This is possible due to the availability of subsequent breath‐hold MR scans, providing information about the intrafractional reproducibility of subsequent breath‐holds. This information is unique and generally not accessible by conventional CT imaging due to the significant imaging dose given for each acquisition. Moreover, the dosimetric consequences of ABC breath‐hold uncertainties were studied. We evaluated robustly optimized IMPT plans which present the state‐of‐the‐art for proton treatments of locally advanced NSCLC patients. The aim of this study was to determine whether the combination of robustly optimized IMPT plans and the delivery under ABC controlled breath‐hold presents a safe approach for PBS lung treatments.

## 2 Methods and materials

### 2.A. Data acquisition

Three‐dimensional T_1_‐weighted MRIs were collected from five representative consented volunteers (3 males, 2 females, age: 25–58 yr, height: 1.65–1.93 m, BMI: 19–29) at the Institute of Cancer Research in London. As described by Kaza et al.*,* a volumetric interpolated breath‐hold examination (VIBE) sequence was used (TR 4 ms, TE 0.93 ms, field‐of‐view 299*399 mm^2^, acquisition matrix 324*576 interpolated, flip angle 8°, acceleration factor GRAPPA3).^14^ The image voxel size was 0.7 × 0.7 × 3.0 mm^3^. MR imaging was performed under ABC controlled breath‐holding, at the 75% threshold of the prior determined maximum deep inspiration volume. The ABC apparatus was modified to be MR‐compatible, as described by Kaza et al.^17^ Only one breath‐hold was required to image the complete lung volume, with a breath‐hold duration of either 22.5 or 25.0 s, according to the number of partitions required. To simulate the fractionated treatment, image acquisition was repeated after 1–4 weeks. During each session, four subsequent MRIs were acquired under ABC controlled breath‐hold, resulting in eight MRIs per volunteer providing both intra‐ and interfraction information about the lung anatomy reproducibility. Imaging was performed using a modified extended wing board for radiotherapy (Oncology Systems Limited, Shropshire, UK).

### 2.B. Deformable image registration

Between the acquired MRIs for each volunteer, a global rigid image registration was applied to account for setup uncertainties. Next, DIR was performed using Mirada's multimodal algorithm RTx v1.6 (Mirada Medical, Oxford, UK). It was done locally on the lung volumes, with a grid resolution of 3.5 × 2.0 × 3.0 mm^3^.

The first MRI of the first simulated fraction was designated as the “planning” MRI. By deforming the planning MRI to the three other MRIs of the same fraction and comparing the resulting deformation vector fields (DVF) DVF_A1A2, DVF_A1A3, and DVF_A1A4, the intrafraction reproducibility of the lung anatomy during breath‐hold was investigated. The interfraction anatomical reproducibility was investigated by comparing the DVF_A1B1, DVF_A1B2, DVF_A1B3, and DVF_A1B4 resulting from deforming the planning MRI of the first simulated fraction to the four MRIs acquired during the second imaging session.

### 2.C. Displacement magnitudes

**Figure 1:**
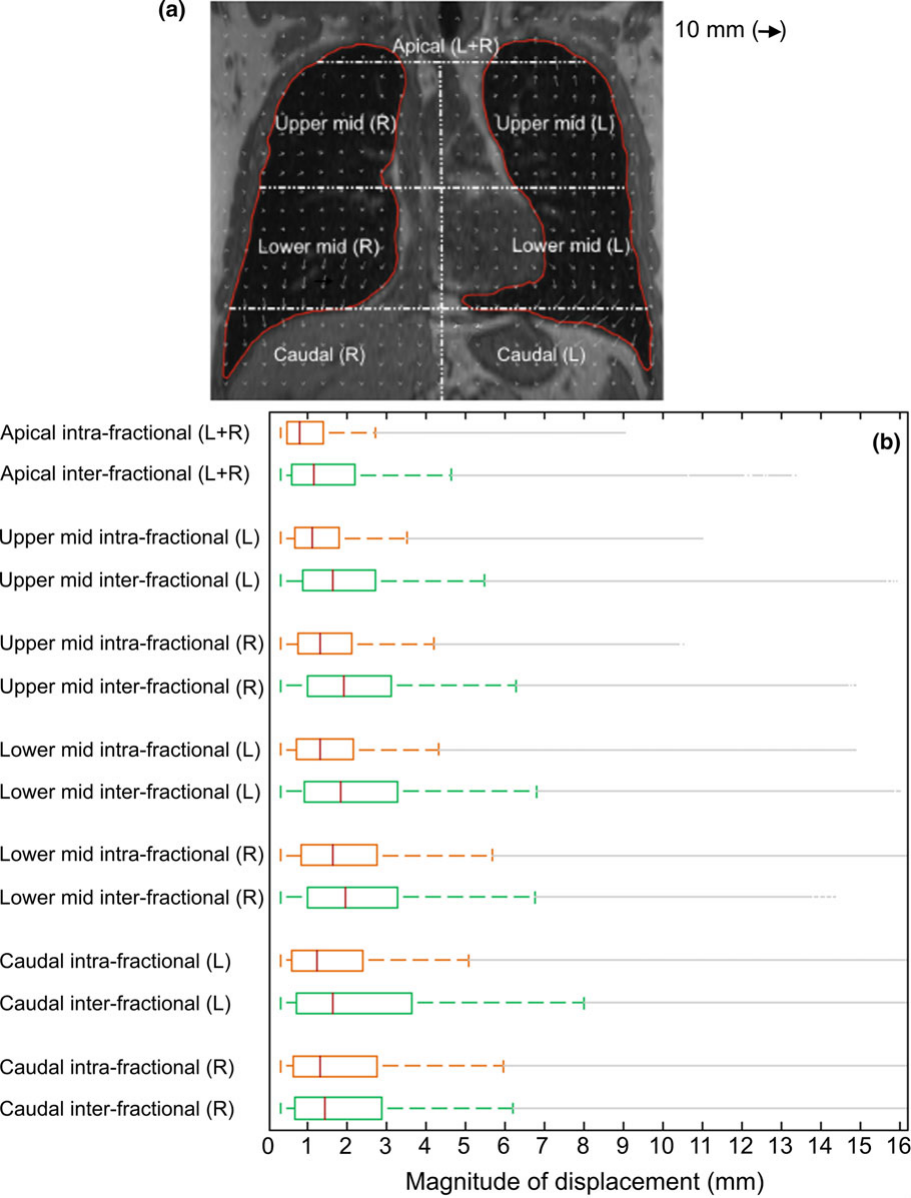
(a) Coronal view of an MR scan overlaid with the deformation vector fields of a deformable image registration between two MRIs for one volunteer. The lung contour was used to guide the deformable registration algorithm. The seven segments that overlap with the lung contour represent the local regions for which the magnitudes of displacement were analyzed. (b) Range of intrafractional (upper boxplots) and interfractional displacements (lower boxplots) for the five volunteers. The middle line in the boxes indicates the median and the bottom and top edges of the boxes the 25^th^ and 75^th^ percentiles. Up to the whiskers the most extreme data points not considered outliers are shown. [Color figure can be viewed at wileyonlinelibrary.com]

Magnitude vectors in 3D were calculated from the DVFs and combined for all seven DVFs in each of the five volunteers using Matlab (v8.3, MathWorks, Natick, MA, USA). To analyze the anatomical displacements on a local level, the lung volumes were divided into seven segments: an apical region, a right/left upper and lower mid region, and a right/left caudal region [Fig. 1(a)].

### 2.D. Deformed CT scans

To generate IMPT/PBS treatment plans and to assess the dosimetric impact of the breath‐hold reproducibility, we generated synthetic CT scans from the MRI data set using the following method:

☐ Breath‐hold CTs of five NSCLC patients (2 males, 3 females, 54–69 yr.) were selected (Table 1). They were matched to respective volunteers based on a visual anatomical match of the lung volumes (Fig. 2).
  **Table 1:** Patient and tumor characteristics

Patient number	Gender	Age	Tumor volume	TNM stage	Original tumor location
1	male	69	38.5 cm^3^	T3N2M0	Left upper lobe
2	male	67	13.5 cm^3^	T4N0M0	Right upper lobe
3	female	56	6.0 cm^3^	T3N2M0	Right middle lobe
4	female	58	22.6 cm^3^	T3N3M0	Left lower lobe
5	female	54	31.6 cm^3^	T3N3M0	Left lower/upper lobe
  **Figure 2:**
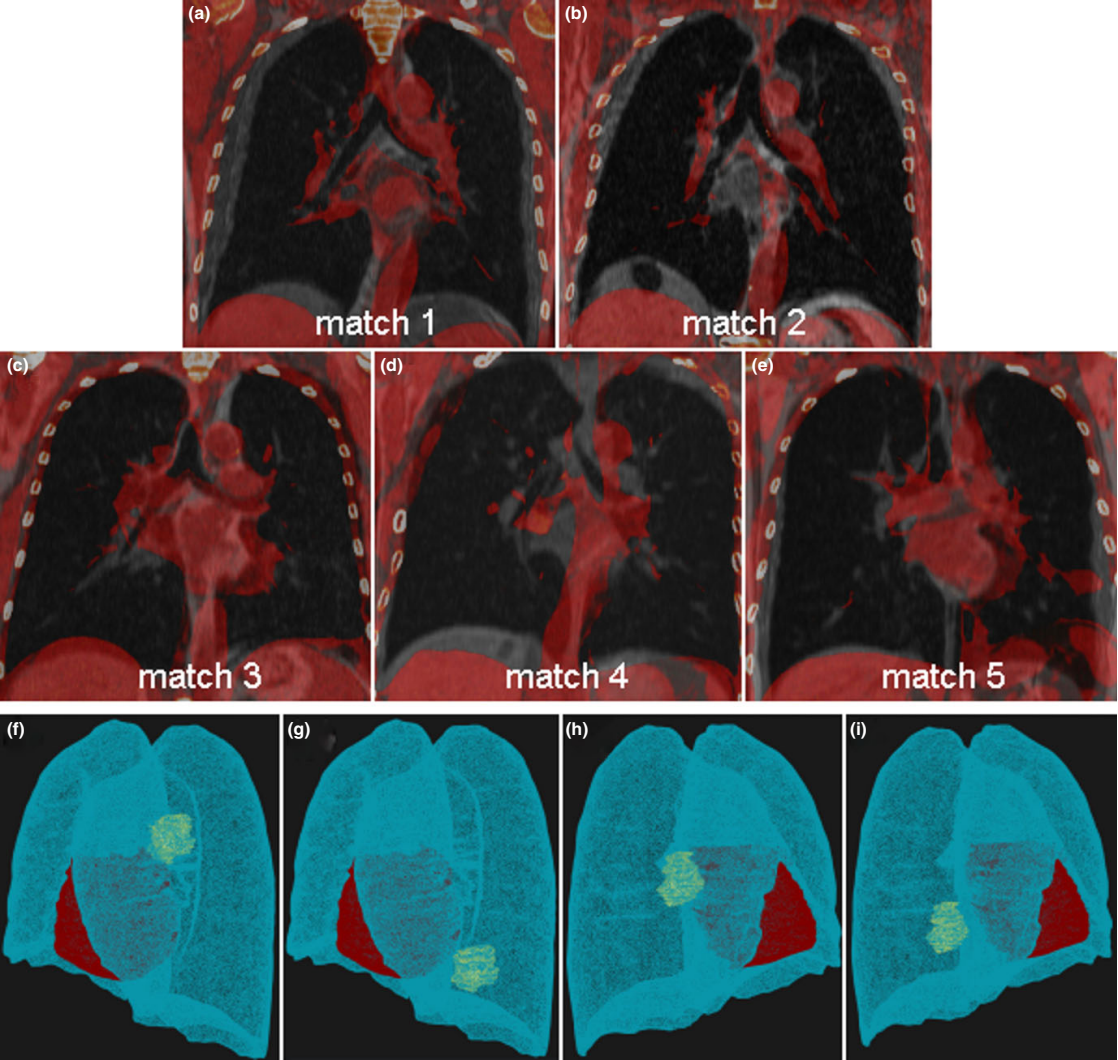
(a)–(e) Fused coronal views of the five patient/volunteer matches. (f)–(i) 3D views of simulated tumor locations for patient number one, including the original tumor location in the left upper lobe (f), left lower lobe (g), right middle lobe (h), and right lower lobe (i). [Color figure can be viewed at wileyonlinelibrary.com]
☐ With Transformix (see elastix toolbox^18^) the CTs were transformed using the DVFs from the matched volunteer. This was performed for all seven DVFs (DVF_A1A2, DVF_A1A3, DVF_A1A4, DVF_A1B1, DVF_A1B2, DVF_A1B3, DVF_A1B4).

This resulted in a set of seven deformed CTs (A2, A3, A4, B1, B2, B3, B4) with different breath‐hold geometries per patient.

### 2.E. Lung tumor modeling

For each patient, we simulated the original tumor configuration plus three other tumor locations. This is to assess the dosimetric impact of the found displacements between breath‐holds for different tumor locations. Involved lymph nodes located in the mediastinum were not included in this simulation, as only the lung anatomy reproducibility is investigated. When the original tumor location was not close to the heart, a fourth simulated tumor location was added. The mean gross target volume (GTV) was 22.4 cm^3^ (range: 6.0–38.5 cm^3^). The GTVs were delineated on the original breath‐hold CTs in RayStation (v.4.99, RaySearch Laboratories Ltd., Stockholm, Sweden). For each simulation the original GTV contour was translated and given a density of 1.05 g/cm^3^ to simulate tumor tissue. The simulated locations included the upper and lower left lung lobes and the middle/upper and lower lobes of the right lung [Figs. 2(f)–2(i)]. GTVs were expanded by 5 mm to create the clinical target volumes (CTV). The CTVs were then deformably warped from the CTs toward the deformed CTs.

### 2.F. Treatment planning

For each of the original and simulated locations (23 cases in total) intensity‐modulated proton therapy plans were created (RayStation v4.99, RaySearch Laboratories Ltd., Stockholm, Sweden). The treatment was given in 25 fractions of 2.4 Gy in 5 weeks to a dose of 60.0 Gy, according to institutional policy. The treatment delivery system was the Proteus^®^ PLUS proton system (IBA, Belgium), including a scanning spot size of 6.5 mm at 70 MeV and 3 mm at 230 MeV (1 sigma). A two‐beam or three‐beam approach was applied depending on the complexity of the case. Beam angles in posterior‐anterior (PA), posterior‐oblique (PO), and lateral (L or R) directions were selected for simulated tumors located posteriorly. Anterior‐posterior (AP), anterior‐oblique (AO), and lateral (L or R) directions were selected for the anteriorly located tumors. For two‐beam configurations only the PA or AP beams and lateral beams were used. To account for setup errors and range uncertainties, minimax robust optimization was applied.^19^, ^20^, ^21^, ^22^ Two treatment plans were created for every case to investigate the effect of ABC breath‐holding uncertainties to treatment plans with different target dose conformities. For the first treatment plan a robustness to 3.0 mm shifts in all directions was aimed at in addition to ± 3% range uncertainties. A robustness to 5.0 mm shifts in all directions was the aim for the second treatment plan together with ±3% range uncertainties. Optimization settings included a set of objectives for the CTV (min dose 59.5 Gy [robust], uniform dose in CTV 60.0 Gy [robust]), and a constraint for the maximum dose (63.0 Gy) within the body. Also, objectives were used for the heart (mean dose, V_5Gy_ [%]), lungs (mean dose and V_20Gy_ [%]), esophagus (V_35Gy_ [%] or D_1cc_[Gy]), and spinal cord (D_0.1cc_[Gy]) to reduce any dose as much as possible, without compromising the robustness of the treatment plan. To stimulate conformal treatment plans, two dose fall‐offs were used. The first dose fall‐off was intended to create a steep high dose gradient (60.0–30.0 Gy) around the CTV and the second dose fall‐off was used to create a gradient from 60.0 to 0.0 Gy dose. A uniform dose grid size was used of 3.0 mm, and a minimum spot weight of 0.011 MU/fraction. During optimization, a constant RBE correction of 1.1 was applied.^23^ After optimization, the robustness was evaluated with an in‐house developed script, applying 14 different setup scenarios accounting for either 3 or 5 mm shifts and ±3% range uncertainties. In total, 28 scenarios were evaluated from which a voxel‐wise minimum dose distribution was calculated.^20^, ^24^ The minimum dose required to be given to 98% of the CTV (D_98%_) was 57.0 Gy for the voxel‐wise minimum dose.

### 2.G. Simulation of ABC controlled treatment delivery

To simulate a treatment delivery during breath‐hold, each plan was split into sub‐plans with delivery duration of 20 s (assumed breath‐hold duration). The number of simulated breath‐holds varied per case. An overview of required breath‐holds is shown in Table 2.

**Table 2:** The number of breath‐holds (20 s. duration) required to deliver one treatment fraction per case and patient for 3 mm and 5 mm setup robustness

	Pt. 1	Pt. 2	Pt. 3	Pt. 4	Pt. 5	Pt. 1	Pt. 2	Pt. 3	Pt. 4	Pt. 5
	Number of breath‐holds									
	3 mm setup robustness					5 mm setup robustness				
Leftupperlobe	5	7	4	7	4	5	7	4	7	4
Leftlowerlobe	–	4	6	6	7	–	4	5	6	7
Rightupperlobe	6	6	5	–	4	8	6	6	–	4
Rightmiddlelobe	6	7	4	4	6	8	7	4	4	7
Rightlowerlobe	6	4	3	7	9	6	6	3	7	8

Next, the subplans were recalculated on the deformed CTs to simulate the dosimetric consequences of using ABC. The full fractionated treatment course was simulated rotating between all available deformed CT scans, starting with the original CT for the first breath‐hold, followed by CT‐A2, then CT‐A3 and CT‐A4. For fractions 1–12 only the deformed CTs with intrafraction uncertainties were considered. For fractions 13–25 deformed CTs with interfraction uncertainties were included, simulating an increase in uncertainties with progressing treatment duration. A schematic overview of the subplan recalculation for one representative patient case is given in Table 3. Dose distributions of the subplans recalculated at the deformed CT scans were mapped to the planning CTs and summed for dosimetric evaluation.

**Table 3:** Overview of the dose recalculation (fully fractionated treatment) for the breath‐hold delivery scenario for one sample case with a three‐beam configuration. Each beam requires two breath‐holds to deliver

		Beam 1	Beam 2	Beam 3
Fraction 1–12	breath‐hold 1	CT A2	CT A4	CT A3
	breath‐hold 2	CT A3	CT A2	CT A4
Fraction 13–25	breath‐hold 1	CT B1	CT B3	CT B1
	breath‐hold 2	CT B2	CT B4	CT B2

### 2.H. Worst‐case scenario simulation

In addition to the described breath‐hold scenario, a worst‐case scenario was simulated for all treatment plans. The whole treatment plan was recalculated on one deformed CT scan deviating the most from the planning CT scan (CT‐B4). This way a systematic error was introduced for all fractions.

### 2.I. Dosimetric evaluation

Several dosimetric parameters were compared between the original treatment and the recalculated plans, according to our institutional policy. Regions of interest that were evaluated included the CTV, heart, lungs (minus GTV), esophagus, and spinal cord. The D_98%_ (Gy) and D_1cc_ (Gy) of the CTV were evaluated. For the heart and lungs, the mean doses were analyzed. Furthermore, the volume receiving 35 Gy and 20 Gy (V_35__Gy_ [%], V_20__Gy_ [%]) to, respectively, the esophagus and lungs was evaluated as well as the dose to 0.1cc (D_0.1cc_[Gy]) of the spinal cord volume.

## 3 Results

### 3.A. Magnitudes of displacements

Figure 1(a) shows the coronal view of a deformable image registration between two MRIs for one volunteer. The displacement magnitudes for all volunteers are shown in Fig. 1(b). The median intrafraction displacement was 1.3 mm and ranged for the segments between 0.8 mm and 1.6 mm. For the midregions, the right lung showed larger intrafraction displacements compared to the left lung (median increased with 0.1–0.3 mm). Interfraction displacements were larger than intrafraction displacements for all regions (median 1.6 mm [range: 1.2–1.9 mm]). Most displacements (75%) remained below 4.0 mm. Maximum displacements (without outliers) reached 5.7 mm intrafractionally and did not exceed 8.0 mm interfractionally. The anatomical reproducibility decreased from the apical regions (maximum displacements <3.0 mm) toward the caudal regions (<8.0 mm maximum displacements).

### 3.B. Dosimetric evaluation of target coverage

**Figure 3:**
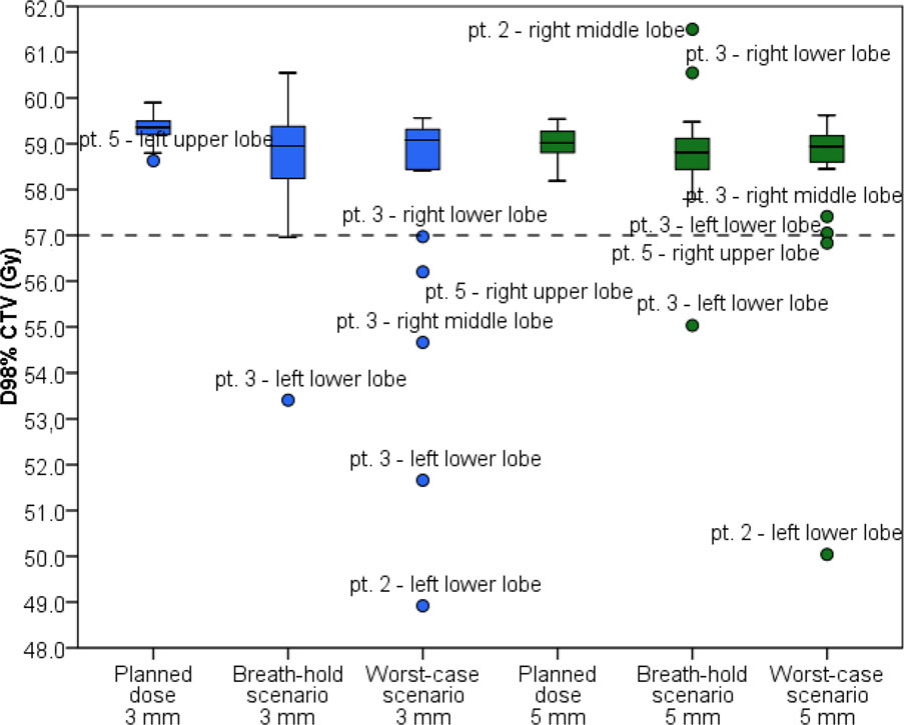
The dose at 98% of the CTV for each simulation and all patients. The tolerance threshold (dotted line) was set to 57.0 Gy of minimum target coverage**.**. [Color figure can be viewed at wileyonlinelibrary.com]

Figure 3 shows the D_98%_ of the CTV for the different simulations and combined for all patients. All nominal plans passed the above described robustness evaluation and were clinically approved based on this evaluation. For the 3 mm setup margin, 22/23 simulations of the breath‐hold scenario achieved the minimally clinically required dose of 57.0 Gy, whereas 18/23 simulations of the worst‐case scenario did. One simulation (pt. 4: right lower lobe) of the breath‐hold scenario showed a D_98%_ of 60.6 Gy, beyond the described dose. For the 5 mm setup margin, the minimum dose of 57.0 Gy that is considered clinically acceptable was achieved for 22/23 simulated breath‐hold scenarios and for 21/23 simulated worst‐case scenarios. Two simulations (pt. 2: right middle lobe, pt. 3: right lower lobe) showed an increase in D_98%_ extending beyond the prescribed dose of 60.0 Gy (61.5 Gy and 60.6 Gy respectively) for the breath‐hold scenario. Table 4 shows the differences in D_98%_ between the planned doses and breath‐hold or worst‐case scenario, for both 3 mm and 5 mm setup robustness settings. For the breath‐hold scenario, the difference in D_98%_ was larger for the middle and lower lobe simulations (median: −0.6 Gy [range: −5.4 to 1.0 Gy]) compared to the upper lobe simulations (median: −0.1 Gy [range: −1.0 to 0.3 Gy]). Also for the 5 mm setup robustness setting, the difference between breath‐hold scenario and planned doses was larger for the middle and lower lobe simulations (median: −0.2 Gy [range: −4.1 to 2.0 Gy]) compared to the upper lobe simulations (median: −0.1 Gy [range: −0.6 to 0.3 Gy]).

**Table 4:** Dosimetric results for CTV and organs at risk shown as differences (mean [range]) between the breath‐hold scenario or worst‐case scenario and the planned dose/volume parameters. Shown for both 3 mm and 5 mm setup robustness settings

	Planned		Planned – Breath‐hold scenario		Planned – Worst‐case scenario	
	Median (range)		Median (range)		Median (range)	
	3 mm setup robustness	5 mm setup robustness	3 mm setup robustness	5 mm setup robustness	3 mm setup robustness	5 mm setup robustness
CTV						
D_98%_ (Gy)	59.4 (58.6–59.9)	59.0 (58.2–59.5)	−0.5 (−5.4–1.0)	−0.1 (−4.1–2.0)	0.0 (−8.0–1.3)	0.0 (−8.8–0.8)
D_1cc_ (Gy)	62.8 (61.7–62.9)	62.3 (61.1–62.9)	0.6 (−0.5–6.1)	0.9 (−0.3–4.6)	0.0 (−5.2–1.8)	0.4 (−0.2–1.7)
Heart						
D_mean_ (Gy)	1.2 (0.1–5.1)	1.4 (0.2–6.0)	0.0 (−0.1–0.3)	0.0 (−0.2–0.2)	0.0 (−0.2–0.2)	0.0 (−0.2–0.2)
Lungs						
D_mean_ (Gy)	4.0 (2.0–7.1)	4.5 (2.3–8.2)	0.0 (−0.3–0.2)	0.0 (−0.3–0.2)	0.0 (−0.3–0.5)	0.0 (−0.9–0.5)
V_20__Gy_ (%)	7.8 (3.9–13.7)	8.8 (4.4–16.7)	0.0 (−0.5–0.3)	0.0 (−0.6–0.3)	0.0 (−1.4–0.6)	0.0 (−1.6–1.2)
Esophagus						
V_35__Gy_ (%)	0.0 (0.0–12.4)	0.0 (0.0–14.3)	0.0 (−0.1–0.3)	0.0 (−0.4–0.5)	0.0 (−0.9–0.9)	0.0 (−0.4–1.1)
Spinal Cord						
D_0.1cc_ (Gy)	0.1 (0.1–15.3)	0.1 (0.0–22.0)	0.0 (−0.1–1.0)	0.0 (−0.4–1.4)	0.0 (−1.9–1.6)	0.0 (−3.2–7.5)

CTV, clinical target volume; D_98%_, dose given to 98% of volume; D_1cc_, dose given to 1 cc of volume; D_mean_, mean dose; V_20_, volume receiving 20 Gy; V_35_, volume receiving 35 Gy; D_0.1cc_, dose given to 0.1cc of volume.

**Figure 4:**
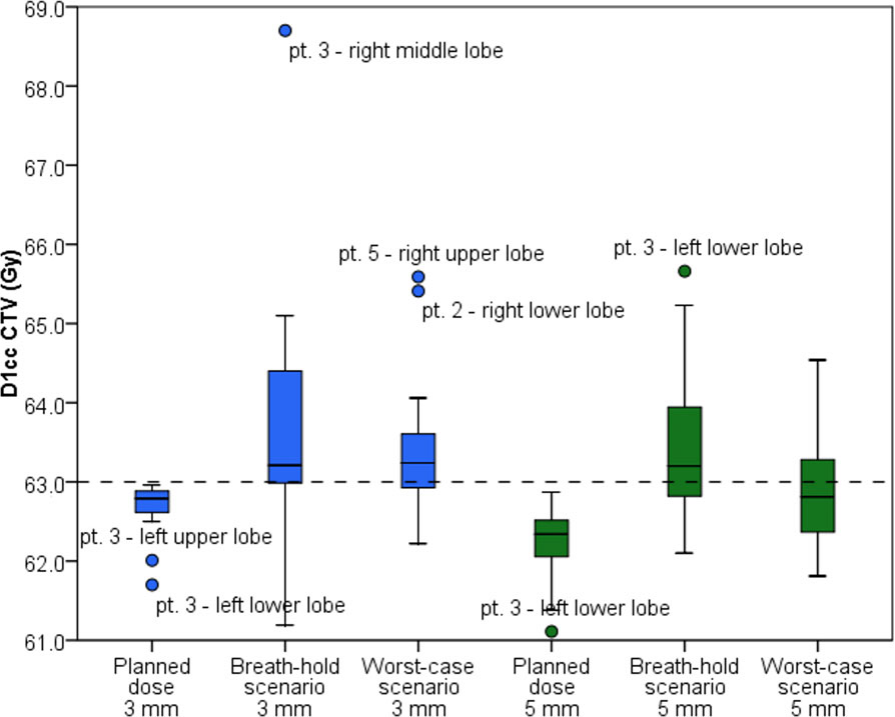
The dose to 1 cc of the CTV for each simulation and all patients. The tolerance threshold of the maximum dose (dotted line) was set to 63.0 Gy. [Color figure can be viewed at wileyonlinelibrary.com]

Figure 4 shows the results of the D_1cc_ to the CTV. For the 3 mm setup robustness setting, the D_1 cc_ increased for 19/23 breath‐hold scenario and 21/23 worst‐case scenario simulations.18 of 23 simulations were beyond the maximum dose of 63.0 Gy for the breath‐hold scenario and 15/23 worst‐case simulations exceeded this threshold. Evaluating the results using the 5 mm setup robustness setting, D_1cc_ increased for 21/23 simulations for both breath‐hold and worst‐case scenarios. For 14/23 breath‐hold scenario and 8/23 worst‐case scenario simulations the increment was beyond the allowed maximum dose of 63.0 Gy. Table 4 shows the differences in D_1cc_ for both robustness settings and the breath‐hold and worst‐case scenario compared to the planned doses. For the breath‐hold scenario and 3 mm setup robustness setting, the median difference in D_1cc_ was smaller for the upper lobe simulations (0.2 Gy [range: −0.4 to 2.2 Gy]) compared to the middle and lower lobe simulations (0.7 Gy [range: −0.5 to 6.1 Gy]). Also for the 5 mm setup robustness setting, the median difference in D_1cc_ was smaller for the upper lobe simulations (0.5 Gy [range: −0.3 to 1.7 Gy]) compared to the middle and lower lobe simulations (1.1 Gy [range: 0.0–4.6 Gy]).

### 3.C. Dosimetric evaluation of organs at risk

Table 4 contains the differences in dose for the heart and lungs for the breath‐hold and worst‐case scenarios compared to the planned doses. Also the volume differences in the esophagus receiving 35.0 Gy and the lungs receiving 20.0 Gy are shown, when comparing the results of the breath‐hold scenario and worst‐case scenario with the planned doses. Both the 3 mm setup and 5 mm setup robustness settings results are shown.

## 4 Discussion

In this study, we investigated the intra‐ and interfractional anatomical reproducibility of ABC controlled breath‐holds. Moreover, we investigated the dosimetric consequences of ABC breath‐hold uncertainties during IMPT/PBS proton treatment in a representative population (23 simulated NSCLC patients). Up to 2 mm median intra‐ and interfraction displacements were found for all lung regions. The maximum displacements increased from the apical regions (~3 mm) toward the caudal regions (~8 mm). Looking at the possible dosimetric impact of the found displacements for 5 mm setup robustness, the evaluation of D_98%_ showed for only one breath‐hold scenario simulation a clinical relevant decrease in target coverage (D_98%_ = 55.0 Gy). Two worst‐case scenario simulations showed a clinical relevant decrease in target coverage (D_98%_ = 50.0 Gy and 56.8 Gy). Two simulations of the breath‐hold scenario showed a relevant increase in the target dose (D_98%_ = 61.5 Gy and 60.6 Gy, D_1cc_ = 65.2 Gy and 64.5 Gy respectively). Twelve more simulations showed a relevant increase in D_1cc_ alone (63.0–65.5 Gy) compared to eight increased D_1cc_ (63.2–64.5 Gy) of the worst‐case scenario simulations. However, this increase was still below a maximum of 110% of the prescribed dose. Evaluating these results with the ones observed for the 3 mm setup robustness, the number of decreased D_98%_ target coverage remained the same for the breath‐hold scenario simulations. It increased from two to four simulations for the worst‐case scenario (D_98%_ = 48.9–56.2 Gy). Only one simulation showed a relevant increase in both D_1cc_ and D_98%._ The number of simulations with increased D_1cc_ was more compared to the 5 mm robustness setting (17/23 breath‐hold scenario simulations and 15/23 worst‐case simulations). We observed that ABC controlled breath‐holding can affect the target coverage by inducing hotspots reducing the target dose homogeneity. This was seen for more simulations using the 3 mm setup robustness setting. Still, the differences between the 3 mm robustness and 5 mm setup robustness setting were relatively small and for most simulations the increased dose heterogeneity stayed within clinical acceptable limits. Moreover, dose differences in the organs at risk were minimal for all scenarios and simulations.

Brock et al. investigated the variability in tumor position and concluded that the intrafraction uncertainties were small (1.5–1.7 mm in all directions). Our finding of a small median displacement for all regions (1.7 mm) confirmed these previous results. For interfraction changes of the lung anatomy, Sarrut et al. found a median displacement of 2.6 mm for eight patients and Brock et al. measured mean displacements of less than 5.1 mm (superior‐inferior).^15^, ^16^ We only observed a small increase in median displacements for interfractional changes that remained under 2.0 mm (0.2–0.6 mm median increase). Dueck et al. investigated the robustness of voluntary breath‐holding for PBS and concluded that small tumors and large baseline shifts are more prone to target coverage loss.^13^ In the current study, we added the effect of intrafraction motion uncertainties and we especially evaluated uncertainties induced by ABC controlled breath‐holding. Furthermore, in contrast with the single‐field uniform dose optimized treatment plans created in the study by Dueck et al. we investigated the dosimetric effect for robustly optimized IMPT treatment plans, which are considered as state‐of‐the‐art for PBS proton therapy and will be the treatment technique used at our facility.

To calculate the displacements between the breath‐hold MRIs, deformable image registration was used. As we applied the multimodal algorithm locally on the lungs, sliding boundaries were not considered. Most of the DIR‐involved uncertainties are considered to be around 2.0 mm,^25^ which is also the median of the displacements found. However, locally we found much larger displacements up to 7.0 mm, which is beyond the range of errors associated with DIR.

Our breath‐hold data were derived from healthy volunteers. We expect that healthy volunteers can hold the breath‐hold more easily than patients. However, ABC controlled breath‐hold as motion mitigation technique will only be applied to patients who are able to at least hold their breath for 20 s. We therefore assume the volunteer data as representative also for the clinical situation.

A limitation of the assessment of anatomical reproducibility based on DVFs obtained from healthy volunteers is that individual tumor movements cannot be considered. Furthermore, involved lymph nodes of the original cases were not included as only the lung anatomy reproducibility is investigated, where the lymph nodes are located in the mediastinum.

In this study we investigated the reproducibility of ABC breath‐holding, where there might be other breath‐holding uncertainties that negatively affect the planned dose distributions. One such phenomenon is breath‐hold drifting. Especially when reproducing a number of breath‐holds this drifting can occur. A limitation of this study is that we did not investigate this effect with the limited number of breath‐holds (four per session), however this would be interesting to investigate for future work. For clinical implementation, image surface scanning could provide a way to monitor the stability of the breath‐hold (eg drifting, slowly exhaling), by imaging the chest surface. This could be in addition to the ABC breath‐hold device, that reproduces the same amount of air that is inhaled.

With our data set we can only approximate a realistic treatment delivery under ABC controlled breath‐hold. A limited number of four intrafraction ABC breath‐hold variations and four interfraction ABC breath‐hold variations for only two simulated fractions were available. A complete course of treatment will generally consist of 25 fractions and the delivery of a single fraction took according to our simulations between three and nine breath‐holds. However, this is the first study evaluating dosimetrically both intra‐ and interfraction uncertainties. The found uncertainties using four breath‐holds and two separate sessions have proven to show only limited dosimetric differences in two different robustness settings and two different scenarios. We can only speculate about the increased dosimetric differences when more than four breath‐holds would have been evaluated per session. By using our data iteratively and in an alternating way we were confident of reflecting the realistic situation with adequate accuracy.

The errors of setup and range during irradiation and the breath‐hold reproducibility were evaluated separately, and we did not evaluate the robustness of the treatment plans to the combined effects of residual setup errors and range errors. However, especially 5 mm setup robustness is quite generous considering a semi‐static treatment situation using ABC breath‐holding after the normal setup positioning using CBCT imaging. We expect that for breath‐holding this added effect of the combination of the residual setup and range error and breath‐holding will be limited.

Finally, the matching of volunteers MRIs with CTs of patients may be improved by use of CT images during repeated breath‐holds to directly create treatment plans and address the dosimetric differences. However, CT image acquisition during eight breath‐holds, of which four repeated breath‐holds per session, is not justifiable for imaging dose reasons. Thus, comprehensive information about repeated breath‐holds could only be obtained with MRI. NSCLC patients would have possibly provided a better resemblance of ABC breath‐hold uncertainties. This is because there might be differences in compliance between healthy and nonhealthy lung tissue, affecting the reproducibility of breath‐hold. However, an addition of eight times MR imaging to the already extensive treatment and imaging schedule of actual patients is considered too much of a time burden.

A prerequisite for small dosimetrical differences between the original and the breath‐hold recalculated plans is the applied robust optimization settings (5 mm or 3 mm shifts and 3% range uncertainties). With especially the 5 mm setup setting, the majority of the ABC breath‐hold uncertainties were accounted for in our study. The question arises what robustness optimization setting would be optimal. A higher robustness inevitably compromises healthy tissue, but ensures adequate target coverage. Where smaller setup robustness settings increase the dose inhomogeneity gradually as observed for the 3 mm setup setting. As future work, we plan to find an optimal cut‐off point between high dose region conformity and robustness of the treatment plan, especially when using ABC controlled breath‐hold. We will assess this cutoff based on risk of compromising target coverage. Patients at higher risk possibly need larger margins; however, patients at lower risk should be planned with smaller margins aiming for more conformity. Moreover, for clinical implementation repeat breath‐hold imaging is recommended to assess the patient specific breath‐hold reproducibility. This to assure that any extremes in reproducibility can be detected and accounted for during the treatment planning.

## Conclusions

The use of ABC for NSCLC patients can be considered safely for IMPT/PBS proton therapy when robust optimization is used during treatment planning. This study indicates that ABC controlled breath‐hold reproducibility uncertainties will not compromise robustly optimized IMPT/PBS plans.

## Conflict of Interest

The authors have no conflicts of interest to disclose.
